# Decision tree for improving the impact of equity-focused public health service delivery and research

**DOI:** 10.3389/frhs.2026.1948535

**Published:** 2026-09-28

**Authors:** Cassandra Lane, Daniel Groombridge, Judith Byaruhanga, Tameka McFadyen, Luke Wolfenden

**Affiliations:** 1 School of Medicine and Public Health, The University of Newcastle, Newcastle, NSW, Australia; 2 Hunter New England Population Health, Hunter New England Local Area Health Service, Newcastle, NSW, Australia; 3 National Centre of Implementation Science, The University of Newcastle, Newcastle, NSW, Australia; 4 Hunter Medical Research Institute, New Lambton Heights, NSW, Australia

**Keywords:** health equity, co-creation, decision tree, health services, implementation science, public health, social determinants of health

## Abstract

**Introduction:**

An equity focus in public health service delivery and research is needed to address persistent health inequities. This requires not only redistribution of health resources and services to prioritised populations, but also attention to the process through which initiatives are selected, designed and implemented, and how these engage with and address the social determinants of health. Applying this in practice can be challenging, and there is limited practical guidance for public health organisations.

**Methods:**

We adapted a decision tree to support equity-focused public health service delivery and research, aimed at practitioners and researchers working to support the co-design and implementation of effective and socio-culturally responsive health initiatives for a known priority population within a defined public health focus area (e.g., tobacco cessation, physical activity, cancer screening). This tool was informed by the literature and iterative development with an interdisciplinary team of public health professionals, implementation scientists, and health equity academics.

**Results:**

The Equity-Focused Decision Tree consists of structured decision points and actions across three steps, underpinned by co-creation principles and ongoing local community engagement. Step 1 – *Identify or co-design and evaluate an initiative*: users integrate empirical and qualitative evidence to a) determine whether any existing initiative is both effective and suitable for the priority population; and b) where none exist, a new initiative is co-designed and evaluated. Step 2 - *Assess evidence-practice gaps*: users determine whether the initiative is being routinely implemented. Step 3 – *Identify implementation strategies*: users identify or co-design evidence- and community-informed implementation strategies that are responsive to the community's needs, values, and context.

**Conclusion:**

This decision tree provides a practical tool to guide public health organisations in applying an equity focus across the process of service delivery and research. It has the potential to advance equity by improving health outcomes for the priority population, and by contributing towards addressing the social determinants of health that underpin persistent health inequities.

## 1 Introduction

Improving public health requires the effective implementation of evidence-based health programs, policies or practices (i.e., initiatives). Various practical tools are available to support public health organisations in this, including a Decision Tree designed to enhance the application of research and research evidence to health service planning and delivery (1). The Decision Tree guides users across three sequential steps, each involving a decision point linked to a suggested service or research action. The process begins with identifying or developing an effective initiative to address an identified health problem, followed by selecting an implementation strategy that supports its uptake and reach, and finally, sustaining and monitoring its implementation. This tool supports population-level health improvement by offering structured guidance in evidence-based initiatives for public health service and research organisations.

Despite global improvements in population health, some population groups continue to experience worse health outcomes compared with others (2, 3), with the greatest burden borne by those adversely affected by multi-level (policy, community, organisational, provider, and individual) social, cultural, structural, political, and commercial determinants of health (hereafter, equity-relevant populations) (4–6). Moreover, efforts to improve whole-of-population health can inadvertently contribute to such disparities by disproportionately improving outcomes among more privileged groups – a phenomenon known as the inequality paradox (7, 8). This was made particularly visible during the COVID-19 pandemic, when health disparities were exacerbated amidst universal public health responses (9) (i.e., uniform models intended for all citizens or members of a broad population group) (10, 11). The urgent need for an equity focus in public health service and research is now widely recognised (12–15).

Policymakers and organisations often seek to advance equity by using a targeted approach, redistributing or tailoring resources and services based on need (selectivism) or specific characteristics of an equity-relevant population (particularism) (10, 11, 16). While this may improve the availability or reach of health promoting infrastructure (17), it is unlikely to be sufficient to address persistent health disparities (6, 18). The 2025 World Report on Social Determinants of Health Equity highlighted that despite improvements in access to healthcare and social services, conditions of daily life, and community environments, inequities persist globally due to the broader “*inequitable political, cultural, social and economic systems that reward the most powerful, to the detriment of the least powerful” (*6). As such, advancing health equity requires attention to these systems and structures through which the determinants of health are produced and maintained (6, 18–24). This distinction is reinforced by philosophical foundations of social equity which emphasise that equity is achieved through both the fair distribution of resources and outcomes (substantive equity) and the fairness of the processes through which decisions are made and outcomes produced (procedural equity) (25, 26).

An equity focus in public health service delivery and research centres co-creation across the process through which evidence-based initiatives are selected, designed, and delivered (6, 14, 27, 28). In practice, this is underpinned by meaningful local community engagement and partnership, characterised by relationship development, building trust, power sharing, reciprocity, and decolonial and antiracist praxes (14, 15, 27–29). Through this, public health service delivery and research can actively contribute to dismantling social and structural determinants of health that shape health outcomes. For example, building trust between providers and equity-relevant populations can improve current and future engagement with health services, while increasing socio-cultural competency and reflexivity among practitioners and researchers strengthens socio-cultural safety and the experiences of those they serve (6, 30–34).

Further, co-creation provides a foundation to generate and integrate socio-cultural knowledge within an initiative and/or its implementation strategies, ensuring these respond to the unique needs, values, and contexts – including the social determinants of health – of equity-relevant populations (35). Socio-culturally responsive initiatives contribute to health improvements for priority populations, while also enhancing initiative sustainment, revitalising and reinforcing culture, and increasing research productivity (6, 30–34). They can also illuminate and interrupt the root causes of inequities which is essential for advancing health equity (6, 36). Intentional efforts to address this and improve health via the intervention or its implementation strategies are needed. An initiative designed specifically “*with, for, and in the community” (*22) is, by definition, developed to be socio-culturally responsive. A universal initiative designed for a broad population rather than with a specific equity-relevant population may be suitable and acceptable for that population, but its socio-cultural responsiveness cannot be assumed. In such cases, socio-cultural responsivity can be addressed at the point of implementation, through distinct strategies, including adaptations to the initiative itself (28, 37).

While there is increasing clarity around the theoretical and methodological considerations required for an equity focus in public health, operationalising this within the real-world systems of public health practice and research remains challenging (6, 29, 38). Various tools and resources have been developed to support efforts aimed at addressing health disparities (39, 40) and improving health equity (23, 28, 41–44). While they provide important conceptual guidance, they remain limited in their practical application for public health organisations (41). Many take the form of conceptual process models and frameworks designed to prompt assessment and action on the underlying causes of inequities (42). For example, the PETAL framework outlines five steps for integrating health equity into Learning Health Systems: prioritise health equity, engage the community, target health disparities, act on the data, and learn and improve (23). Similarly, Kilbourne et al.'s disparities research agenda describes a phased research approach from detecting disparities, to understanding disparities (identifying determinants), to designing initiatives to reduce or eliminate disparities (39). Other tools focus on guiding research and evaluation (39, 40) such as the decision tree developed by Chinman et al (40), which supports researchers in applying implementation science designs to health disparities research (40).

In addition, what is also needed is practical guidance to support public health organisations in applying an equity focus across health service design, delivery and research. The Decision Tree offers a useful structure for addressing this when informed by the equity principles described above. We therefore adapted the Decision Tree with the aim to produce an Equity-Focused Decision Tree to support public health organisations in the selection or design, and implementation of initiatives to address a public health focus area for one or more prioritised equity-relevant populations.

## 2 Methods

The Equity-Focused Decision Tree was developed in response to practical needs and experience within an Australian public health unit, organisational and government policy directions, and the published literature (9, 41, 45–48). Its development followed a triangulation-informed approach, integrating multiple forms of evidence and conceptual knowledge, and translating these into adaptations of the original Decision Tree through an iterative process. Practical and effective tools can be developed through approaches that bring together multiple sources of knowledge to inform tool development and refinement (49). Development was undertaken by an interdisciplinary team of public health professionals, implementation scientists, and health equity academics with diverse professional, cultural, and experiential perspectives. Several team members also brought lived community experience relevant to populations experiencing health inequities. These perspectives informed discussions about how health initiatives may be experienced, designed, and implemented differently across equity-relevant populations. We recognise that researchers and practitioners may simultaneously occupy multiple and intersecting positions (50), which informed the adaptations. While these perspectives contributed to the development of the tool, they are not considered a substitute for engagement with the communities with whom the tool is intended to be applied, as discussed further in the Discussion.

The development process began with two authors (CL, DG), who were co-leading a cultural and equity network within a NSW Local Health District, supporting implementation of population health initiatives for children and young people in community settings, and leading research with equity-relevant populations. Through this work and ongoing discussions, they identified that, in order to improve health outcomes equitably, actions involved in selecting, designing, and implementing health initiatives could differ when working with equity-relevant populations and when equity was an explicit focus. The lead author (CL) subsequently adapted the original Decision Tree to explore how these actions could be incorporated. An initial visual model was developed based on these discussions and existing knowledge of the health equity literature. The initial adaptation retained the three-step structure of the original Decision Tree but incorporated two key changes: (i) co-creation as an overarching principle; and (ii) decision logic adapted to distinguish between initiatives that were universal and those that had been co-designed with a priority population (described further in the Results). The initial version was discussed with DG and considered to provide a useful representation of the conceptual adaptations that had emerged through their discussions, practice, acquired knowledge from delivering health initiatives with priority populations, knowledge generated through research and community consultation with priority populations, and lived experience (such as DG being a First Nations person). This included learnings from Indigenous-specific research, including a cultural evaluation of a school-based nutrition initiative by the authors (37).

A targeted literature review was undertaken to identify existing health equity frameworks, tools and implementation guidance relevant to the emerging components of the adapted model. The lead authors examined these, including those identified in several evidence syntheses, including the National Collaborating Centre for Determinants of Health 2023 rapid review of health equity frameworks, guidance documents, and tools (41); Gustafson's scoping review of equity-focused implementation theories, models, and frameworks in healthcare (29); and the University of Victoria Health Equity Tools inventory (43). In addition, they reviewed the literature on policy approaches, including Francis-Oliviero et al.'s scoping review of proportionate universalism (47) and Carey et al's framework for its application (11). This review identified a need for more practice-oriented guidance, while also informing the identification and synthesis of best-practice actions, principles, and resources to support equity-focused public health service delivery and research.

The emerging tool was subsequently shared with the broader interdisciplinary team and progressively refined. The team engaged in several cycles of drafting, critical reflection, targeted literature review, and collaborative dialogue (35, 51). Targeted literature searches examined specific components of the emerging tool, including best-practice approaches to co-design and community engagement, equity-focused implementation science, and approaches to addressing social and structural determinants of health. Team members reviewed proposed decision points and actions and raised questions or identified aspects of the decision logic requiring clarification. Where questions or disagreement arose, these were addressed through discussion and, where needed, further examination of the literature and clarification of the underlying decision logic. These discussions resulted in the iterative refinement and clarification of the decision points and their accompanying actions. This process supported refinement of the tool to align with theory, empirical evidence, practical experience in public health research and working with communities, and reflexive insights.

## 3 Results

The Equity-Focused Decision Tree (see Figure 1) consists of suggested actions and structured decision points to support equity-focused public health service delivery and research in response to a defined public health topic area (e.g., tobacco cessation, physical activity, cancer screening) and a prioritised population. The tool guides users across three sequential steps, underpinned by ongoing community engagement.

**Figure 1:**
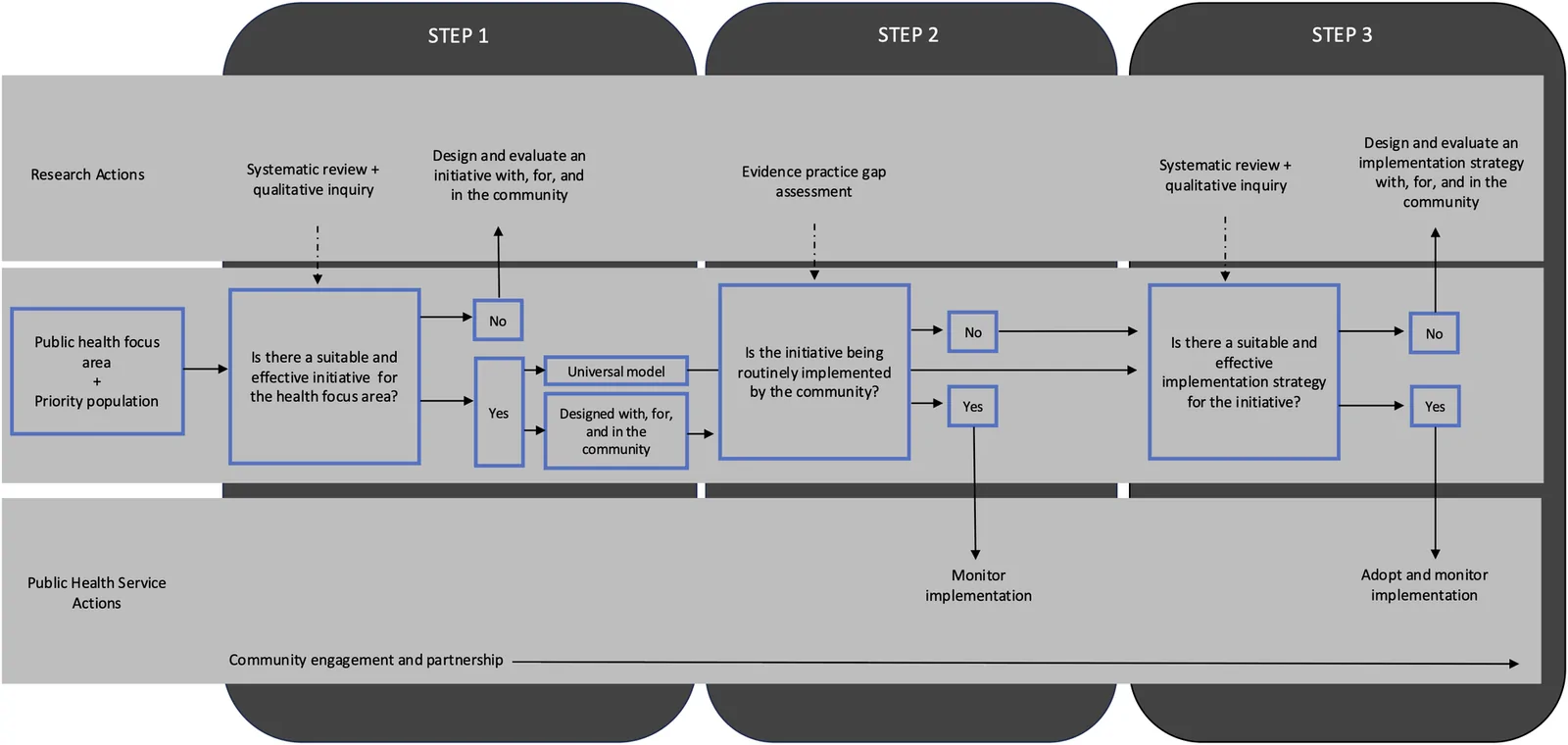
Equity-Focused Decision Tree for public health service delivery and research. Adapted from the original Decision Tree by Wolfenden et al (2020) [1].

### 3.1 Step 1. Identify or co-design and evaluate an initiative

Users first seek evidence to determine whether there is a suitable and effective (evidence-based) existing initiative to address a defined health topic area for a prioritised population. Effectiveness refers to evidence that the initiative produces the intended outcomes, while suitability refers to whether it is feasible to deliver, acceptable (i.e., satisfactory and appealing), and appropriate (relevant and compatible) (52, 53). Importantly, an initiative can be evidence-based yet still unsuitable for the prioritised population or context; therefore, both effectiveness and suitability must be established. To assess these dimensions, both academic evidence (systematic reviews), and local evidence (qualitative inquiry from community and public health practice) are synthesised for decision making.

#### 3.1.1 Method of assessment

Systematic reviews are recommended to assess evidence of effectiveness (54). This starts with a search for existing reviews (e.g., via Cochrane Library) that provide recent and relevant evidence on the effectiveness of the initiative in improving the health outcome of interest or its determinants for the priority population (55). If none are identified, users can conduct a systematic review following the Preferred Reporting Items for Systematic Reviews and Meta Analysis-Equity (PRISMA-E) (56) statement, and with technical support and guidance from best practice tools such as the JBI manual for evidence synthesis (57) and the Cochrane handbook (58).

Local evidence is essential for assessing whether an initiative is suitable. This can include the perspective of the priority population as well as local health services and practitioners who often support delivery of initiatives and are embedded within the communities they serve (21, 59). Local evidence may be drawn from multiple qualitative sources, including academic and grey literature, reviews of qualitative studies, and direct inquiry (e.g., interviews, workshops, feedback activities, fieldwork, or observations) to capture lived experience and local perspectives (28). Qualitative inquiry should prioritise interpretive and reflexive methods that attend to power and the situated nature of knowledge (60, 61). Users may refer to resources on equity-focused qualitative research (61, 62) and decolonising qualitative research (63).

Through assessing suitability, the fit between the public health topic area and the situational needs and priorities of the community may become apparent (64, 65). Ideally, the topic has been identified from within the community or confirmed as warranting attention (66). Where there is misalignment, constructive strategies include reassessing initial assumptions, adjusting priorities to align with community values, and redefining the topic and its potential impacts in ways that resonate more strongly with lived realities (67). Ongoing community engagement is key to supporting trust and shared understanding (68), and practical guidance is available to support meaningful and inclusive engagement (68, 69).

Evidence can be synthesised for decision making through a process of triangulation, using multiple perspectives to arrive at a more reliable, and accurate understanding of whether an initiative is effective and suitable (70, 71). This process should engage community members as partners in reviewing, interpreting, and contextualising academic evidence, so that local insights and lived experiences inform decision making (28). The Health Equity Interactive Systems Framework describes a 'Synthesis System' for this purpose, emphasising the active engagement of both academic and community interest holders in compiling and summarising pertinent information (28).

#### 3.1.2 Decision points

##### 3.1.2.1 An effective and suitable initiative is identified

Where an effective and suitable initiative is identified, users then consider whether it was designed with, for, and in the community and is therefore more likely to be responsive to the needs, values, and contexts of the prioritised population. If so, users proceed to Step 2 to assess whether it is being routinely implemented for optimal impact.

If the identified initiative has not been co-designed – often the case for universal models intended for a broader reference population, or targeted models that have lacked in meaningful community engagement - equity-focused action shifts to the point of implementation, consistent with the principles described above. Users proceed directly to Step 3, where co-design is applied to select, design, and/or adapt socio-culturally responsive implementation strategies.

##### 3.1.2.2 An effective and suitable initiative is not identified or there is insufficient evidence

Where an effective and suitable initiative is not identified or there is insufficient evidence, users conduct further research, starting with the co-design of an initiative with the priority population. This draws on the meaningful community engagement already embedded in the broader co-creation process, alongside participatory methods, to ensure the initiative is socio-culturally responsive and empowers communities, while strengthening trust with public health organisations and staff – all of which enhances the likelihood of effectiveness and equity impact (6, 11, 27, 66). This process may also uncover existing community strengths or actions that can be leveraged in addressing the health topic.

Next, the co-designed initiative is evaluated to test its effectiveness. Equity research values study designs that centre partnerships, reciprocity, community empowerment, and socio-culturally responsive ways of knowing, being, and doing (28). Randomised and controlled trials (RCTs) are often used to establish effectiveness, and users can refer to Jull et al.'s conceptual framework for designing a health equity relevant randomised and controlled trial (RCT) (55). Where appropriate, users may consider pragmatic trial designs (e.g., stepped-wedge or quasi-experimental) alongside participatory and qualitative research (4, 64, 72, 73). Additional outcomes, such as suitability (as described in Step 1) and community-defined measures of success should also be considered (15).

### 3.2 Step 2. Assess for evidence practice gaps

Users proceed to assess whether the co-designed initiative is being implemented in practice by the prioritised population, identifying whether there are gaps that may require investment in strategies to improve implementation of the initiative.

#### 3.2.1 Methods of assessment

Common outcomes for assessing implementation include acceptability, adoption, appropriateness, feasibility, fidelity, cost, reach and sustainability (53). Assessment of evidence-practice gaps can draw on direct or proxy measures of such indicators, using readily available data sources or by establishing new sources of data where required (1, 74). Where feasible, data should be collected as close as possible to the context in which implementation occurs, including through engagement with the population, organisations, and settings involved in implementation, to improve the relevance and contextual validity of the assessment (74).

For implementation research regarding equity-relevant populations, community engagement - ideally through leadership by, or co-production with, the priority population - should inform assessment processes, including the identification of relevant and important indicators, appropriate and feasible data collection methods, and the interpretation and communication of findings (4, 74, 75). Shelton et al. (4). also emphasise the importance of transparent metrics and data sharing (or open access), as well as the use of mixed methods and qualitative methods to amplify the voices of equity-relevant populations.

Users may also apply an intersectional lens to explore whether implementation gaps exist for sub-groups within the prioritised population (e.g., by socially stratifying factor/s such as gender, sexual orientation, or socioeconomic status) (76, 77). Resources are available to support this, including the Intersectionality Toolbox developed by Sabik et al., which guides researchers in applying an intersectional lens (77), and the Health Equity Impact Assessment (HEIA), a systematic process to assess how initiatives may affect health equity across population groups and to inform actions to mitigate identified impacts (78, 79). Although disaggregated data based on select characteristic/s are central to monitoring inequalities (80), it should serve as a foundation for creating change (81) rather than a demonstration of disparity (81, 82). This is particularly important for Indigenous populations and communities (self-identified differently across global regions), where extractive data practices and a lack of reciprocity have historically eroded trust (83).

#### 3.2.2 Decision points

##### 3.2.2.1 The initiative is routinely and appropriately implemented

If the evidence-practice gap assessment indicates that the initiative is being routinely and appropriately implemented, no further investment in enhancing implementation is required. However, a monitoring strategy should be established to assess ongoing sustainment and to identify any emerging gaps that may require action. Sustainment ensures that the health benefits are achieved over the long term, preserves rapport with communities, and protects the initial investment of resources (84). Sustainment has been highlighted as especially important for equity-relevant pouplations (85), without which public health organisations risk losing community trust (84). Sustainment strategies may be required, for example, through investment in materials and/or fostering partnerships that can be used in the absence of ongoing resources (86).

##### 3.2.2.2 The initiative is not routinely implemented

If an evidence-practice gap is identified, strategies are required to support adequate implementation, and users proceed to Step 3.

### 3.3 Step 3. Identify implementation strategies

Users arrive at Step 3 when the initiative identified in Step 1 was not co-designed, or because an evidence-practice gap was identified in Step 2. Like Step 1, users proceed to assess for an implementation strategy that is suitable and effective for the prioritised population.

#### 3.3.1 Methods of assessment

Implementation Science methods should be used to identify an implementation strategy (or package of strategies) that is effective and suitable for the priority population. This involves first identifying the factors (barriers and facilitators) influencing implementation of the initiative and then identifying implementation strategies specifically to address these factors.

Theoretical implementation science frameworks are used to delineate factors and then guide the systematic linkage with appropriate strategies. The Consolidated Framework for Implementation Research (CFIR) is widely used and includes five domains: innovation (the initiative itself), outer setting, inner setting, individuals: roles and characteristics, and implementation process. The CFIR has been adapted to incorporate equity considerations across its domains, including power dimensions (87) and race-consciousness (88). The Health Equity Implementation Framework (HEIF) includes an additional three domains that capture key implementation factors for equity-relevant populations: cultural factors, patient-provider interactions, and social contexts that shape and reinforce inequities (75). Other frameworks, like the CFIR, can be adapted to include these equity-relevant domains (89) and this has been successfully done to advance implementation research for equity-relevant populations (88–92). The HEIF has also been incorporated into a recent meta-framework, the IM4Equity, to centre community-engaged approaches and equity within implementation processes (93).

Implementation factors can be examined through systematic reviews that synthesise comparable evidence, alongside local evidence from the community perspective (knowledge and experiences of priority population members) and health service perspective (knowledge and experience from staff within a local health unit) (21, 28, 59). With an equity focus, assessments should prioritise the HEIF equity domains, including the historical, social, and structural contexts that shape health inequities at organisational or system, implementer, and recipient levels. This includes examining how and why equity-relevant populations may not be aware of, able to access, or benefit from an initiative (94). These considerations are often overlooked in conventional assessments of implementation factors (4, 89).

Once determinants are identified, they can be systematically linked to strategies intended to address them. A wide range of implementation strategies are available (95) and many may be suitable or adaptable to address identified barriers (more on adaptation described below). However, existing taxonomies do not fully account for the social and structural implementation barriers faced by many equity-relevant populations (i.e., HEIF equity domains) (4, 21, 96). Implementation strategies recommended for equity include: critical perspectives, community-defined evidence, building equitable teams, resource and power sharing, increasing trust and partnerships, building capacity and resources within communities, raising awareness of and addressing biases among implementers and systems, dismantling power differentials, enhancing cultural competence, advocacy and allyship, and adaptations to the initiative itself (21, 22, 28, 96, 97). For Indigenous communities, implementation strategies should also consider cultural safety, trusted relationships, Indigenous workforce capacity, and community control (96, 98, 99).

After potential strategies are identified, the methods described in Step 1 are followed, assessing evidence of their effectiveness and suitability for the priority population. Briefly, systematic reviews can be used to assess whether implementation strategies improve implementation outcomes and address equity-relevant implementation determinants. Where possible, assessments should also examine whether implementation outcomes are distributed equitably across population groups. The EquIR provides guidance for incorporating equity considerations across implementation outcomes (44). Where no relevant review exists, users may consider conducting one. Qualitative inquiry is also used to assess whether strategies are feasible within existing resources and infrastructure and whether they are responsive to the needs, values, and contexts of communities. As in Step 1, local evidence includes both practice evidence from health services and delivery providers (external support) and community evidence from recipients and implementers. Evidence can then be synthesised to inform decision making.

#### 3.3.2 Decision points

##### 3.3.2.1 An effective and responsive implementation strategy is identified

Users adopt the implementation strategy and monitor implementation sustainment, providing additional support if implementation falters. Again, sustainment is critical for achieving long-term benefit, protecting initial investment, and maintaining community trust.

##### 3.3.2.2 An effective and responsive implementation strategy is not identified

Where no effective or suitable implementation strategy is identified, further research is required to support implementation strategy selection. This process follows the research considerations outlined in Step 1, starting with co-creation of an implementation strategy with the priority population and followed with an implementation trial to test its effectiveness. In many cases, existing implementation strategies can be adapted such that they are responsive to the needs, values, and contexts of the priority populations (22). Many established adaptation frameworks are based on participatory and co-design research principles, making them well aligned for equity research. For example, the Making Optimal Decisions for Intervention Flexibility during Implementation (MODIFI 100) framework offers a human-centred approach, guiding users to co-design adaptations that are grounded in locally-relevant evidence.

## 4 Discussion and conclusion

Substantive health and societal benefits could accrue with greater investment in more equitable implementation of effective public health interventions (101, 102). We present a decision tree that may be useful for other public health practitioners and researchers working to promote and support the implementation of effective and socio-culturally responsive health initiatives, with an explicit equity focus. This resource responds to multiple calls for practical guidance in advancing equity-focused public health (9, 41, 47, 48, 103) by offering clear, actionable steps to support health improvement and reduce the risk of harms (104). An important next step will be to explore and document its application in real-world settings, to assess its feasibility, acceptability, utility, and adaptability (including demonstrated use in diverse contexts) for public health practitioners, researchers, and communities.

While the Equity-Focused Decision Tree provides a structured foundation to guide decision-making, it is intended as a template rather than a prescriptive tool. Its application and/or adaptation should be undertaken collaboratively with equity-relevant communities, with the decision points, methods, and actions accommodating the unique needs, values, and contexts of distinct equity-relevant populations and settings. The Tree represents broad equity principles that serve as a starting point for decision-making, while the work itself should be informed by the communities with whom the work is undertaken. In practice, the Tree could be adapted to ensure that it prioritises and reflects socio-culturally grounded perspectives and methodologies. For example, the Equity-based Framework for Implementation Research (EquIR; an equity-focused implementation framework) has been adapted for Māori, the Indigenous peoples of Aotearoa New Zealand, through a co-creation process that centred Indigenous knowledge systems and priorities (105).

Conceptually, the Tree contributes to the field of implementation science. Implementation is widely recognised as a critical opportunity to advance equity (4, 11, 16, 22, 94, 106), with the potential to strengthen the uptake, fidelity, efficiency, and overall effectiveness of an initiative by responding directly to the needs, values, and contexts of equity-relevant populations (106). The Equity-Focused Decision Tree makes this opportunity explicit and actionable, demonstrating how equity considerations can be systematically embedded within implementation processes. The application of this decision tree also has the potential to contribute to addressing key gaps in the field (4, 22), particularly around implementation strategies that address the social and structural implementation barriers faced by many equity-relevant populations (e.g., HEIF equity domains. 75) Shelton et al., have highlighted how “*little research has examined feasibility, effectiveness, uptake, acceptability of strategies or mechanisms through which they work with an eye towards equity”* (4). By guiding the co-design and evaluation of implementation strategies that are focused specifically on equity-relevant domains (Step 3), this tool offers a structured approach with the potential to generate this much-needed evidence. Future research should examine whether and how application of the Equity-Focused Decision Tree and/or its actions supports the development of equity-focused strategies, and, ultimately, equity-related health outcomes.

Another contribution of this work relates to the operationalisation of equitable public health service delivery models. Public health organisations are increasingly required to balance the pursuit of population-level impact with a deliberate focus on prioritised populations. Proportionate universalism is often recommended (19), however, there remains limited guidance on how to use this approach in practice, particularly how universal initiatives can be augmented to achieve more equitable outcomes (9, 47, 103). This Tree illustrates how universal initiatives could be retained for population-level impact, while being augmented at the point of implementation through targeted, responsive strategies co-created with a priority population. Importantly, the value of this approach extends beyond the selection of a health service delivery model to the processes through which decisions are made and enacted. Co-creation supports the development of socio-culturally responsive implementation strategies (35) – thereby enhancing effectiveness, equity, and attention to the social determinants of health (6, 30–34). In this way, the Equity-Focused Decision Tree can guide the application of proportionate universalism to achieve more equitable population-level outcomes.

We also acknowledge potential limitations. In terms of scope, the tool may not apply equally to all types of public health initiatives. For example, emergency or crisis situations may necessitate urgent universal models, whereas this Tree may be particularly beneficial during recovery phases or in contexts that allow for deliberate planning. It was also developed by a team working in Australia, and although they drew on diverse experiences across other global contexts, its applicability and adaptability across different countries and health systems will need to be examined. In terms of methods, the Tree was informed by reviews of existing frameworks and tools and targeted reviews of literature relevant to specific components; however, it was not developed through a formal systematic review and no predefined criteria were established for the identification and selection of supporting frameworks or recommendations. Further, although the Tree was developed collaboratively, no formal consensus method (e.g., Delphi process or nominal group technique) was used. The Equity-Focused Decision Tree would benefit from further evaluation and refinement through application in diverse public health and community contexts.

Finally, it is important to acknowledge that this tool is an adaptation of the original Decision Tree grounded largely in biomedical and behavioural science paradigms, reflecting a degree of methodological conformity in both its overall structure and component steps. While we have drawn on social science and equity theories and frameworks to adapt the tool, its capacity to support more transformative change is likely limited. Advancing equity has been argued to require approaches that extend beyond adaptation within existing paradigms, toward decolonial perspectives that interrogate and challenge the underlying epistemological foundations of research and practice (15, 24, 107). For example, greater alignment with decolonial approaches would involve community identification of the public health focus area itself, thereby redistributing agenda-setting authority to equity-relevant communities (107). While Step 1 encourages consideration of whether the pre-defined public health topic area aligns with community-defined needs, and recommends strategies to address misalignment, this structure may still reflect underlying power asymmetries (107).

Despite these limitations, as others have noted, “*There is no right path to advancing health equity. It is not necessary, or always possible, to have all the answers before embarking on this work*” (41). In this context, the Equity-Focused Decision Tree may represent a practical step forward. It offers a pragmatic and actionable approach that – if deemed suitable by users – may work within current public health systems to support more deliberate efforts to advance health equity. Its application may also help to foster greater critical awareness and reflexivity among public health organisations and researchers – capacities that are essential for enabling the broader transformative change required for equity (15, 24, 107). Systematic application of the steps and its principles using approaches that seek to learn from and share the knowledge generated, may be a promising path to more equitable population health outcomes (108).

## Data Availability

The original contributions presented in the study are included in the article. Further inquiries can be directed to the corresponding author.
